# Sample size formula for joint modelling of longitudinal and time-to-event data in clinical trials

**DOI:** 10.1186/1745-6215-14-S1-O108

**Published:** 2013-11-29

**Authors:** Matthew Powney, Paula Williamson, Ruwanthi Kolamunnage-Dona

**Affiliations:** 1 University of Liverpool, Liverpool, UK

## 

In many clinical trials, longitudinal or repeated measures data is collected in order to monitor the patient's progress over time towards a clinical end-point. One method of accounting for the two outcomes is by using joint modelling methods. However, little work has been done on sample size calculations for studies which use joint modelling of longitudinal and time-to-event data to establish treatment effects. We propose a sample size formula based on the random effects formulation of the joint model. The method calculates the sample size and power based on the number of longitudinal time points by extending the Schoenfeld [1] approach to sample size for the proportional hazards model. We derive the required number of events using the score statistic [2] based on the random effects joint model [3].

## References

[B1] Schoenfeld D Sample Size Formula for the Proportional-Hazards Regression Model Biometrics 1983 14 499 503 10.2307/2531021 6354290

[B2] Rao CR Linear Statistical Inference and It's Applications 1973 New York: Wiley

[B3] Henderson R Diggle P Dobson A Joint Modelling of longitudinal measurements and event time data Biostatistics 2000 14 4 465 480 10.1093/biostatistics/1.4.465 12933568

